# Reply to Shafaei B. Comment on “Giraldo-Ocampo et al. B Cell Subsets in Colombian Adults with Predominantly Antibody Deficiencies, Bronchiectasis or Recurrent Pneumonia. *Adv. Respir. Med.* 2022, *90*, 254–266”

**DOI:** 10.3390/arm93030016

**Published:** 2025-06-09

**Authors:** Andres F. Zea-Vera

**Affiliations:** Departamento de Microbiología, Universidad del Valle, Cl. 4b #36-00, Cali 760032, Colombia; andres.zea@correounivalle.edu.co

I am writing in response to the thoughtful observations [1] raised by a reader concerning a perceived “controversy” regarding the reported frequency of CD21low B cells in our article titled “B Cell Subsets in Colombian Adults with Predominantly Antibody Deficiencies, Bronchiectasis or Recurrent Pneumonia” [2].

In our manuscript, we described five CVID patients who had more than 1% CD19+ B cells, and among them, we stated the following: “Three out of these five CVID patients also had an increase in CD21low B cells’ absolute frequency”. This statement referred specifically to the absolute count of CD21low B cells, not the percentage. As shown in the Table 1, CVID-2, CVID-4, and CVID-6 had elevated absolute CD21low B cell counts. However, only CVID-4 and CVID-6 had percentages above 10%, which, according to the EUROclass classification [3], is the threshold to define the CD21low phenotype.

Thus, although three patients (CVID-2, 4, and 6) had high absolute numbers of CD21low B cells, only two of them (CVID-4 and CVID-6) met the >10% relative frequency criterion necessary to be classified as CD21low in the EUROclass system. We deeply appreciate the reader’s interest and the opportunity to clarify this point. Such discussions only strengthen the scientific community’s collective understanding, and we remain grateful for the engagement with our work.

## Figures and Tables

**Table 1:** CVID patients with CD19+ B cells >1%.

	% Frec.CD21lo.CD19	Abso.CD21lo/µL	Euroclass
CVID-2	4.49	19	smB+21norm
CVID-4	27.3	25	smB-Trnorm, smB-21lo
CVID-5	0.99	1	smB+21norm
CVID-6	19.9	162	smB-Trnorm, smB-21lo
CVID-8	1.29	5	smB+21norm
